# A Medical Retrospect of 1913: The Lessons of Experience

**Published:** 1914-01-03

**Authors:** 


### A MEDICAL RETROSPECT OF 1913.

### The Lessons of Experience.

THE last twelvemonth has been one of the most disturbed and momentous that the profession of medicine in this kingdom has ever hitherto experienced. Compared with the effects of the National Insurance Act all other questions of medical policy and politics fade into insignificance; and there is every reason to suppose that the future results of that particular piece of legislation will be more far-reaching even than those with which medical men are only too familiar already.

It is true that the year has borne its full quota of professional researches and discoveries, though it has not been signally distinguished in this respect over most of its recent forerunners. These we do not propose to summarise here. The most important of them have already received passing attention in our columns, and fuller accounts of them are available year by year in the *Medical Annual*, and publications of similar scope. The greatest event of the year, apart from the panel controversy, was the seventeenth International Congress of Medicine, held in London in the summer. This also was sufficiently described at the time; memorable as the Congress was, it is now ancient history, and has little, if any, calculable bearing upon the future.

### THE FUTURE OF NATIONAL INSURANCE.

With the Insurance Act the position is radically different. The profession has as yet only reached the fringe of the vortex which the Act is creating. Momentous as the disturbance has already been, it seems likely that in the not distant future the full force of the whirlpool will be encountered; and whether the profession of medicine will sink or swim may depend very largely upon its corporate determination and preparedness for the struggle. In these circumstances a brief breathing space in which to look back on the immediate past is a useful measure; for the lessons of history are a valuable guide to those who would mould the course of coming events. The mistakes of the past cannot, of course, be rectified now, but to review them may help towards avoiding similar errors should similar circumstances occur again, as they not improbably may. Let us, therefore, look back for a moment at the controversies of a year ago, and let us especially note wherein our own views proved right or wrong, our own comments on the unfolding drama justified or not by the dénouement. Where we prophesied correctly, let us not adopt an I-told-you-so attitude: where we did not, let us seek out the causes of our failure.

Rather more than a year ago the Chancellor of the Exchequer, after a desperate struggle with the British Medical Association over the capitation fee to be allotted for panel service, offered that substantial advance upon his original terms which stands to-day as the recognised tariff. The offer was a compromise representing approximately a half-way house between the terms originally offered and those demanded by the British Medical Association. On more than one occasion the opinion was plainly expressed in these pages that the profession would be wise to negotiate upon the basis of this compromise: not because the terms themselves were just and adequate, but because it was quite clear the Chancellor could not possibly offer better without serious loss of prestige. The Association, by a proportion of six to one, or thereabouts, rejected these terms, and implicitly declared for a fight to a finish.

### THE DOWNFALL OF THE B.M.A.

Relying on the common sense and loyalty to each other and to the Association of the members of the profession, we then fully expected to see them victorious. But the Chancellor had his back to the wall, and was practically fighting for his political existence. All the arts of party warfare were employed to cajole and intimidate the doctors; and no sooner was the battle joined than the majority of them in a panic turned tail and fled; nay, even deserted to the adversary. This astonishing and pitiable exhibition of cowardice and pusillanimity amazed not only ourselves but the entire lay public as well. Treachery within the camp there was, no doubt, but not enough to account for the immediate rout of the army when victory was within sight and only the most elementary cohesion and loyalty was needed to secure it. How far the issue is to be regarded as a precedent is still uncertain. At first sight the rational conclusion would needs be that a learned profession which has not got sense enough to follow the example of unskilled labourers by sticking together in an industrial dispute is hopelessly unfitted ever to undertake the conduct of one again. But in arrest of this it has to be admitted that a year ago the political experience of the profession was *nil*, and that even among dock labourers and coal-miners the trades union movement took considerable time to get into full swing.

What of the signs of the times, then? Is the medical profession showing any signs that it is learning wisdom from its defeat? At first, after the downfall, there was little that could be construed thus. The leaders complained of the followers who promised to support them and then did nothing of the sort. The rank and file abused the leaders for bad generalship. The British Medical Association appeared to have surrendered at discretion, and to be dominated by the mutineers. A score or more of dissentient organisations sprang up in opposition, most of them petty, isolated, powerless. Gradually order has begun to evolve out of chaos, though even yet there is not nearly enough appreciation by the members of the profession at large of the fact that a crisis looms ahead, graver and more menacing than that which overwhelmed them a year ago.

The British Medical Association, it is true, is still practically in a state of paralysis on this question; and there is little sign that those who direct its policy have any clear conception of what the problems of the future are, or how they are to be met. The Association has suffered a good many defections from its ranks, and is likely to suffer more through the recent increase in the rate of subscription. It is endeavouring to recast its constitution in some such way as will allow of less cumbrous action than at present. In a word, it displays signs of restlessness in its torpor, and may possibly wake up some day.

Six months ago the National Medical Guild was the most persistent and energetic of the various new bodies which came into existence after the downfall of the British Medical Association. For some time the Guild enjoyed the hospitality of these columns wherewith to expound its policy. Briefly, the leading idea of its organisers was to secure recognition as a trades union in order to secure the advantages of the Act of 1906, which places trades unions above the law of the land and gives their funds immunity from attachment for various crimes committed by their agents or officers. The ardour and perseverance with which this plan was advocated by the Guild were remarkable; but the prejudice of the profession was apparently aroused by the term "trades union," and the response to the Guild's programme was very limited. Nor was statesmanship conspicuous among the Guild's chief officials, and eventually it became obvious that salvation for the profession of medicine was more likely to be found in other directions. The present position of the Guild was fully indicated in the report of its meeting at Hammersmith published in our last issue.

The latest, and in many ways the most important and outstanding development of the situation is the federation together of numerous societies into the National Medical Union. These constituent bodies are united in the determination of their members not to take up panel work in the present conditions which surround it. The Union has just issued its confession of faith, in the shape of four tenets, printed in full on p. 380. Comments upon them will be found prefixed to the four articles of the policy, and we commend the whole matter most earnestly to the consideration not only of the medical professions, but also to that of all thoughtful citizens interested in the future of the public health of the insured classes.

### The Tuberculosis Problem.

In other respects, the further expansion of the State campaign against tuberculosis has been a feature of the year. The great bone of contention as to the relative value of tuberculin and sanatoria is still dividing the experts. Perhaps it would be correct to say that the year has seen some searching criticisms of tuberculin which have not been answered with quite the same authority and success as in former years. Nevertheless, tuberculin dispensaries have sprung up in abundance and vast numbers of the tuberculous are receiving treatment there. Of these institutions, too, critics are not lacking; but sufficient time has hardly yet elapsed for any final estimate of the work which is done. Certainly, the faith which has been freely pinned to tuberculin dispensaries has not up to the present been justified by results achieved and proved beyond all questioning.

### The Medical Year in Germany and the United States.

Abroad there are two developments of the year which are of outstanding importance. One of these is the long struggle in Germany between the Leipzig League (of doctors) and the approved societies. Just at the very end of the year a peace of some kind was arrived at; although the exact details have not yet come to hand, it is evident that the Leipzig League held such strong cards, and played them so well, that it has achieved a great and material victory in the fight. Especially noteworthy is the limitation of each practitioner's panel list to 1,350 insured patients. An exceptionally conscientious panel practitioner recently related his experience of panel practice to the writer. He has one thousand insured patients, and he finds that he has to see at least thirty of them every day—sometimes more—at his house, and to pay on an average six or seven visits. He finds that he cannot accept more on his list without neglecting the interests of those who are sick; so, being a conscientious man, he limits his list voluntarily to one thousand. What a commentary is this upon the panel lists of four, five, and six thousand, not uncommon in London at this day.

The other epoch-making development, to which attention was drawn in The Hospital at the time (November 15, p. 158), may possibly prove to be of far-reaching importance to the medical profession, to medical education and research, and to institutional interests. It is the provision at Johns Hopkins Hospital, of Baltimore, of such large salaries for the professors of clinical subjects that the best type of consulting physician or surgeon will consent to a condition prohibiting private practice altogether. From all points of view, except that of the private patients who can afford to pay specialists' fees, the scheme is admirable; and it is conceivable that eventually it may come to be the prevalent mode, if not, indeed, universal. But at present the idea is visionary as far as this country is concerned, for reasons of finance; and will in all probability remain so for many years to come. Nevertheless, the working of the scheme at Baltimore will be watched with close interest and sympathy. It does not do to forget that the Johns Hopkins foundations, in a short career of not much over twenty years or so, have proved themselves over and over again to be the advance guard of progress. Sir William Osler, who left his professorship there in order to take up that at Oxford University, may be expected to keep us posted in the results of the experiment, which is just such a project as will appeal to his practical mind and scholarly genius.

